# Fibro-Cystic Tumor of Inferior Maxilla—Excision of Right Lateral Half of That Bone

**Published:** 1872-01

**Authors:** 


### ARTICLE VII.

### *Fibro-Cystic Tumor of Inferior Maxilla—Excision of Right Lateral Half of that Bone.*

This patient, Miss P. L——, aged 20, is from Hartsville Tennessee, and is acccompanied by her most excellent and kind physician, Dr. Dyer. She is the subject, as you see, of a large tumor, which occupies the right lateral half of the inferior jaw bone. Three years since it was first noticed as a small globular swelling, unaccompanied with pain or tenderness, just anterior to the angle of the jaw. It grew gradually, until it had attained the size of a goose-egg, when Miss L—— came to the city to place herself under my treatment. I was absent at the time of her visit. Prof. Nichol examined the growth, and reports that the tumor occupied the posterior third of the body of the bone, the ramus not involved; smooth, hard, and seeming to spring from the bone, and about the size of a goose-egg.

The lady was advised to return to her home and come back in a month, when I would have returned. Unfortunately, she listened to the advice of some person, and placed herself in the hands of a notorious quack styling himself, "Indian Doctor," who promised to remove the growth by his wonderful blood-purifier. As a matter of course, she obtained no benefit from the medicine, the tumor pursuing the even tenor of its way, without let or hindrance, until now it has assumed huge proportions. It is now, as you see, as large as the two fists of a man, extending from near the symphisis of the chin to the articulation, reaching far down in the neck, and encroaching high up on the superior maxillary. The skin covering the growth is natural in appearance; the surface of the tumor pretty regularly globular, and smooth, with points here and there, which give evidence, by touch, of the presence of fluid; while at other parts it is hard and firm, as if its walls were formed of bone. It is not painful, nor tender upon handling. The general health of the patient is excellent.

I regard this tumor as benignant, and of the class Fibro-Cystic, which commenced its growth in the cancellous structure of the bone, and has broken up the bones by its gradual increase. If an operation had been done in May, 1870, when the patient first came to see me, it is probable that the cyst might have been opened, and a pledget of lint placed in it, so as to excite granulations, and bring about its contraction and complete cure. Nothing but an excision of nearly one-half of the lower jaw will do now. Excision of a portion of the jaw is an American operation—in fact, a

Tennessee operation. It was was done by Dr. Deaderick, of Athens, East Tennessee, in 1810.

To remove the jaw, with the tumor attached, I will commence by pushing this sharp-pointed bistoury to the bone, just below the red border of the lip, and making a perpendicular incision to the base of the jaw, then under its lower border to its angle, thence up over the posterior part of the angle to a part half an inch below the zygoma. This large semi-circular flap is to be dissected from the tumor, and turned upwards; the lower part, that which is encroaching on the neck, is likewise to be uncovered by the knife. Then, the canine tooth having been extracted, the bone is to be divided at that point, by the saw and bone forceps. The soft parts, on the inner side, are to be separated from the bone as far as the angle, when it (the bone) is to drawn outward, and the knife kept constantly in contact with it, until the coronoid process is freed, and the articulation is opened, when the remaining attachments can be divided with impunity, and then the whole mass separated. Of course the facial, submental and transverse facial arteries, will be divided, and give rise to very free hemorrhage; and unless the precausions mentioned above be observed, of keeping the knife close to the bone, from the angle to the articulation, there is danger of wounding the internal maxillary artery; the hemorrhage from them can be arrested, for the time, by the little pincettes, and the ligatures applied when the operation is finished. A piece of lint, saturated with a solution of carbolic acid, should be applied to the cut surface in the mouth, and the flaps brought back to their place, and fastened with the silver suture.

For a few days, the swelling will we so great as to interfere with the process of swallowing. In one case, I had to resort to the stomach-tube, in order to get nourishment into my patient's mouth, for more than a week. This, however, was an exceptional case. Generally, a sufficient amount of liquid can be swallowed to sustain life until the swelling subsides.

(The operation was then performed. It was found tha the bone had been broken in two parts by the growth of the tumor, above the angle, and the coronoid process and articulating head of the bone had to be removed separately, which was effected by grasping these parts by the Ferguson's lion-toothed forceps, and dissecting them out cautiously. The tumor, upon examination, proved to be cys ic, its walls formed of dense fibrous structure, strengthened here and there by plates of bones which had been thrust out by the growth, having dense, strong septa passing through it at various points, forming smaller cysts, each containing a fluid, very much like the white of egg. The patient is fast recovering, is able to be up, and the deformity resulting from the operation is very slight.)—*St. Vincent's Hospital Reports.—Nashville Journal of Medicine and Surgery.*

---

